# Editorial

**Published:** 1860-01

**Authors:** 


### EDITORIAL.

In issuing the first number of a new Medical Periodical, we have only a few words of explanation to offer.

Chicago has not only become a great commercial metropolis, but also a prominent medical centre, to which a large part of the profession in the North-West, look both for their education and their medical literature. From a somewhat extended intercourse with this part of the profession, we are satisfied that they desire a medical journal, which, in addition to furnishing them, from month to month, with a fair supply of valuable practical and scientific matter, shall possess sufficient independence in its editorial management, to convey impartial and reliable information in regard to all the medical institutions existing among us; and sufficient liberality to open its columns to well written articles from respectable members of the profession, whether the sentiments they contain accord, in all respects, with those entertained by the editors or not. In other words, they want a journal conducted with energy, independence, and liberality; embracing as its paramount object the upbuilding of the profession by the advancement of its practical, scientific, social, and educational interests. With some degree of reluctance we have undertaken the task of supplying such a journal. But having put our hands "to the plough," we shall not look backward. On the contrary, we shall spare neither time, labor, nor money, to make the *Examiner* all that its readers could reasonably ask. We have secured the aid of a good list of collaborators, embracing some of the most eminent practitioners in three or four States. Our home resources for matter of both practical and scientific interest, are ample. We not only have now two Medical Colleges in active operation in this city, but we have three Public Hospitals, two Dispensaries, one Charitable Eye and Ear Infirmary, and two well organized Medical Societies. From all these we shall gather more or less matter for the entertainment and instruction of our readers. We also earnestly solicit contributions from members of the profession generally.

In pecuniary matters, our past experience has satisfied us, that payment in advance is the only correct policy.

It is certainly more easy, and far more pleasant, to pay promptly *two* dollars per annum, than to pay eight or ten dollars at the end of four or five years.

We have issued this, the first number, one month in advance of its date, to give those who wish to subscribe, time to send in their names and remittances before the issue of the second number, which will be due on the first of February.

To the editorial fraternity, we would extend a friendly greeting, and respectfully ask them to place the *Examiner* on their list of exchanges.

---

### CHICAGO COLLEGE OF PHARMACY.

The introductory exercises of this Institution were inaugurated in Bryant & Stratton's Commercial College, with a general introductory lecture, by Prof. J. H. Rauch, M. D., which was listened to with interest by an audience of ladies, physicians, and students. The subject of the address was the History of Pharmacy, and displayed much research and labor in its composition.

E. A. S.

### TRANSACTIONS OF STATE SOCIETY.

A desire to give some idea of the progress of our Western medical literature, we trust, will be considered a sufficient motive for having devoted to the Transactions more space than generally it is advisable to appropriate to a bibliographical notice. We hope that the Abstract will furnish to the profession an idea of their value, and those who consider themselves entitled to a copy, and do not receive one, will please remember that a resolution was adopted by the Society at its last meeting, by which those members only are entitled to the Transactions who have paid the assessment of three dollars.

All those who have thus responded, have been supplied, and to others of the profession who will remit to the Treasurer, Dr. J. W. Freer, the same will be immediately sent. Also, there are a number of copies of the Transactions for the years 1856, '57, and '58, still in the hands of the Secretary, that, when bound, would constitute a handsome volume, and a valuable addition to the library of any physician. E. A. S.

---

### MEDICAL DEPARTMENT OF LIND UNIVERSITY.

The first annual course of instruction in this institution was commenced on the evening of the 10th of October last. The College Hall was closely crowded by an audience composed of professional men, students, and intelligent citizens of both sexes. The exercises were opened with prayer by the Rev. J. A. Wight, after which the general introductory lecture was delivered by Prof. N. S. Davis. As this lecture is published in full in the present number of the *Examiner*, every reader will judge of its merits for himself. It was listened to by the audience with evident interest and pleasure. At the close of the exercises, the audience was invited to examine the laboratory, museum, and other rooms provided for the accommodation of the institution. The number of the regularly matriculated students at that time was 18, which has since been increased to 26, namely, 14 in the Junior, and 12 in the Senior Departments. This is regarded by the friends of the institution, and the Faculty, as a very satisfactory beginning. Dr. Titus Deville, the Professor of Anatomy, did not arrive from Europe until two or three weeks after the commencement of the term. On his arrival, he gave a general introductory to his course of lectures, which was listened to by a large and highly delighted audience. The lecture was elegantly written, and highly appropriate to the occasion.

This institution has thus fairly commenced its work, and taken its place among the permanent medical schools of our country.

---

### RUSH MEDICAL COLLEGE.

The regular term of lectures in this institution commenced on the first Tuesday in November. The general introductory lecture was delivered by Prof. J. A. Allen, formerly of Michigan, and was listened to with pleasure by the audience. The lecture was beautifully written, but in sentiment advocated a strict adherence to the established system of medical college instruction, with all its defects and absurdities. The number of students does not vary materially from that of former years; and, we are glad to remark, an increase of one professorship, and the advantage that the students now possess of listening to a few lectures upon the important branch of Microscopy and its teachings by the Professor of Surgical Anatomy. As trivial as these changes and additions are, we cannot but regard them as an earnest of more important ones in the future. And, that the organization of a new and rival school, can but stimulate to further exertion, and a consequent increase of the number of students. E. A. S.

